# In-depth phenotypic characterization of multicellular tumor spheroids: Effects of 5-Fluorouracil

**DOI:** 10.1371/journal.pone.0188100

**Published:** 2017-11-15

**Authors:** Angélique Virgone-Carlotta, Manon Lemasson, Hichem C. Mertani, Jean-Jacques Diaz, Sylvain Monnier, Thomas Dehoux, Hélène Delanoë-Ayari, Charlotte Rivière, Jean-Paul Rieu

**Affiliations:** 1 Univ Lyon, Université Claude Bernard Lyon 1, CNRS, Institut Lumière Matière, Villeurbanne, France; 2 Centre de Recherche en Cancérologie de Lyon, UMR INSERM 1052 CNRS Centre Léon Bérard, Lyon, France; University of Navarra, SPAIN

## Abstract

MultiCellular Tumor Spheroids (MCTS), which mimic the 3-Dimensional (3D) organization of a tumor, are considered as better models than conventional cultures in 2-Dimensions (2D) to study cancer cell biology and to evaluate the response to chemotherapeutic drugs. A real time and quantitative follow-up of MCTS with simple and robust readouts to evaluate drug efficacy is still missing. Here, we evaluate the chemotherapeutic drug 5-Fluorouracil (5-FU) response on the growth and integrity of MCTS two days after treatment of MCTS and for three colorectal carcinoma cell lines with different cohesive properties (HT29, HCT116 and SW480). We found different sensitivity to 5-FU for the three CRC cell lines, ranging from high (SW480), intermediate (HCT116) and low (HT29) and the same hierarchy of CRC cell lines sensitivity is conserved in 2D. We also evidence that 5-FU has a strong impact on spheroid cohesion, with the apparition of a number of single detaching cells from the spheroid in a 5-FU dose- and cell line-dependent manner. We propose an innovative methodology for the chemosensitivity evaluation in 3D MCTS that recapitulates and regionalizes the 5-FU-induced changes within MCTS over time. These robust phenotypic read-outs could be easily scalable for high-throughput drug screening that may include different types of cancer cells to take into account tumor heterogeneity and resistance to treatment.

## Introduction

Significant improvements have been made in cancer therapy but there is still a need for real time quantification of the progression of various biological processes (differentiation, proliferation, invasion, death…) on fresh living samples and for innovative drug screening methodologies. Functional analysis of cancer cells survival in response to chemotherapeutic agents could be used to adjust the treatment strategy and to predict the therapeutic outcome. Traditional two-dimensional (2D) cell-based assays are commonly employed to evaluate drug sensitivity patterns [1]. However, results from such 2D platforms are often very different from the *in vivo* as cell interactions are restrained to neighbouring flat cells and underlying extracellular matrix [2,3]. Three dimensional (3D) cells aggregates, called Multicellular Tumor Spheroids (MCTS), recapitulate with better fidelity the organization of cells found *in vivo* and represent a recognized non-vascularized tumor model [4]. It is now well acknowledged that MCTS are apt *in vitro* models for drug screening in the field of oncology and especially for the translation of anticancer therapeutics to the clinic, as it mimics not only 3D cell-cell and cell-matrix interactions, but also the biochemical environment of the in vivo tumour mass [4]. However, even though biologists have been using MCTS since more than 40 years in laboratories [5–7], MCTS are just beginning to be routinely employed for drug screening [8]. Recent studies showing that chemotherapeutic molecules identified in 3D models are distinct from those found in 2D [9] have renewed the interest of MCTS in drug screening platforms to better predict *in vivo* efficacy of drug candidates [10]. The slow emergence of MCTS model, despite its non-ambiguous relevance, arises from the increased costs and complex preparation compared to its 2D counterparts, and more importantly, from the lack of standard protocol for the quantification of drug potency.

Many complex methodologies have been developed to assess treatment efficacy in 3D by following the number of proliferating cells using the analysis of frozen or paraffin-embedded slices [11] or tricky selective dissociation protocols [12] and FACS sorting [13,14]. The dynamic and non-destructive follow-up of MCTS over time include daily analysis of spheroid volume growth extracted from standard phase contrast microscopy [15] or more global analysis of metabolic activity within spheroids such as the Acid Phosphatase Assay (APH assay [8]) and Tetrazolium-based assay (MTT assay, [16]). Many studies are also focusing on genes and proteins expression modifications in MCTS and its implication on cell resistance to drug treatment [17,18] with few correlating these modifications to phenotyping changes within spheroids [16,19]. Overall, there is still a crucial need for innovative methodologies that would allow the quantification of drug efficacy on 3D models with simple and easy readouts to accelerate the translation of novel therapies from the lab to the clinic [20].

The aim of this study is to systematically evaluate the dose response to 5-Fluorouracil (5-FU) during the first 48h of treatment in 3D colorectal carcinoma (CRC) models and to establish robust phenotypic read-outs for MCTS response to treatment using microscopies widely available in research labs. 5-FU is one of the most commonly used drug in chemotherapy against CRC. It is an anti-metabolite inducing DNA damage, and is widely given in first-line of treatment in many types of solid cancers [21]. As it is well known that cells at different tumour stages are exhibiting different sensitivity to drugs, we have chosen to compare three classical CRC cell lines: HT29, HCT116 and SW480.

We evidence that 5-FU has a strong impact on spheroid cohesion, with the apparition of a number of single cells detaching from the spheroid in a 5-FU dose- and cell line-dependent manner. We describe a methodology based on a new 3D-IC_50_ calculated from spheroid residual and cohesive core size after transfer in fresh medium to new wells. We show that the disintegration degree of peripheral cells and the nature of detached cells after drug is a valuable independent measure of drug efficacy. Using this simple methodology, we can quantify indirectly the MCTS cohesion that is an indicator of cell sensitivity to 5-FU. We propose it could be easily scalable for high-throughput drug screening that may include different types of cancer cells to take into account tumor heterogeneity and resistance to treatments.

## Materials and methods

### Colorectal cancer lines

HT29 colorectal adenocarcinoma (HTB-38) cell line, HCT116 colorectal carcinoma (CCL-247) cell line, and SW480 (CCL-228) adenocarcinoma cell line were purchased from the American Type Culture Collection (ATCC, Virginia, USA). All cell lines were cultured in Dulbecco’s Modified Eagle’s medium (DMEM-Glutamax), supplemented with 10% of heat-inactivated fetal bovine serum (FBS; Sigma, St. Louis, Missouri, US), 100 units/100 μg of penicillin/streptomycin.

### Cell culture in two dimensions (2D)

The three cell lines are grown in petri dishes and placed in an incubator at 37°C and 5% CO_2_ in an atmosphere saturated with H_2_O. The culture medium is changed regularly, and the cell passage is carried out at 70% confluency. Trypsin (0,25%, Gibco) is used to detach the cells from the substrate.

### 3D cell model: MCTS formation

MCTS are formed in Ultra Low Attachment (ULA) 96 wells Round-Bottom plate treated to be non-adhesive (Greiner bio-one), thereby avoiding cell-substrate attachment. The cells are trypsinized, a coloration of dead cells with trypan blue (0,4%, Sigma) is assessed and cells are counted using a Malassez grid in order to obtain 2400 cells per milliliter. This concentration of cells (*i*.*e*., 480 cells per well in a volume of 200 μL) is chosen in order to obtain a single spheroid per well, with a spheroid diameter at the end of the experimentation not exceeding 500 μm. The plate is centrifuged for 5 minutes at 1200 g at room temperature to initiate the formation of spheroids. The plate is placed in the incubator under agitation at 37°C and 5% CO_2_ during all the experiment. At the end of the first day after seeding, 100 μL of culture is added to ensure proper 3D growth.

### Spreading assay

Multi-wells plates (μ-Slide 8 Wells ibiTreat, ibidi, Germany) are covered with 100 μL of a 0,5 mg/ml collagen type I (rat tail, Gibco) solution at 4°C and then kept at 37°C for 1 hour. MCTS are transferred on these plates, placed in a stage incubator at 37°C, 5% CO2, 100% humidity (INUBG2E-GSI-BP-H2-SL, Tokai Hit) and recorded during 50 hours with a confocal microscope (Leica SP5) using XYZt scans.

### 5-FU treatment

5-Fluorouracil (5-FU) was kindly provided by Centre Léon Bérard (Lyon, France) purchased at Sanofi-Aventis, stored at room temperature at 384 mM in sterile water and diluted in cell culture medium. 5-FU acts during the S phase of the cell cycle inhibiting DNA synthesis by restricting availability of thymidylate and can also inhibit RNA synthesis [14]. MCTS are formed following the usual protocol of 3D cell culture described above. After two days, they are treated with 5-FU at various concentrations: 1, 2, 5, 10, 25, 50, 100 and 300 μM. For control spheroids, only the culture medium is renewed. MCTS are followed during 48h post 5-FU. Twelve samples (N = 12) are made for each concentration after checking the homogeneity of spheroid size in each wells.

### Spheroids transfer protocol

A ring of detaching cells appears spontaneously after one-day of 5-FU treatment. By transferring spheroids into new well plates, we eliminate mechanically this uncohesive peripheral cell layer. We thus obtain an unbiased area of the spheroid cohesive core. For each transfer, the drug and culture medium are renewed.

### Live and dead assay

Spheroids are labeled directly in 96-well plates after removing the maximum of culture medium using calcein (Thermofisher, 1 uM) and Propidium Iodide (PI; Sigma, 2mg/ml) diluted in sterile PBS 1X (Gibco). Calcein labels viable cells cytoplasm in green, PI labels nuclei of dead cells in red showing red dots intercalating DNA (PI+ cells). In order to avoid cell dispersion especially in the loose peripheral layer all around spheroids for the labeling, we add 50 μL of a 2% low temperature gelling agarose solution prior to the live and dead labeling. The double staining was performed during 20 minutes at 37°C before epifluorescence microscope analysis.

### Phase-contrast follow-up of MCTS core diameter

3D MCTS photographs are taken with an inverted microscope (Leica DMIRB) in phase contrast inside the 96-well plates at 24h, 32h and 48h after 5-FU exposure. MCTS are transferred to new 96-well plates at 24h and 48h and photographs are taken before and after transfers. A segmentation based on a sobel threshold is made for each spheroid to isolate properly the core using ImageJ free software. From these binary images, the spheroids are fitted with an ellipse and major (L_M_) and minor (L_m_) axes are calculated using ImageJ “Analyze Particles” plugins. From this, a mean diameter is calculated (D = L_m_+L_M_/2). The volume V is then extrapolated making the assumption that the spheroids are spherical $\left(V=\;\frac{4}{3}\pi R^{3}\right)$.

### 2D-cellular impedance assay by real-time cell analysis (RTCA)

The RTCA impedance assay (xCELLigence RTCA, ACEA Biosciences) is a noninvasive real-time monitoring of cell proliferation, cell size and morphology, continuously providing data over days. Three separate 16-well plates with electrodes embedded are controlled and monitored in parallel. The instrument is placed in a standard CO_2_ cell culture incubator and is powered and controlled via a cable connected to the control unit housed outside the incubator. The functional unit of a cellular impedance assay is a set of gold microelectrodes fused to the bottom surface of a microtiter plate well (E-Plates®). The impedance magnitude is dependent on the number of cells, the size and shape of the cells, and the cell-substrate attachment quality and is reported as a Cell Index parameter. Cells are seeded at a concentration of 5000 cells per well in culture medium. 5-FU is added 48 hours after cells seeding, by removal of culture medium and replacing by 5-FU diluted in culture medium at concentrations of 1, 2, 5, 10, 25, 50 and 100 μM (200 μL per well). Cell proliferation was monitored during 50 hours.

### 2D videomicroscopy analyses

2D cell proliferation is followed with an inverted microscope (Leica DMIRB) using time lapse phase contrast imaging in controlled CO_2_, temperature and humidity parameters during 120 hours in 24-wells plate (Corning). The medium volume per well is 1 mL. 5-FU is added 48h post incubation at concentrations of 1, 2, 5, 10, 25, 50 and 100 μM. Control spheroids are supplemented with new culture medium at the same time and the kinetics of 2D cell proliferation is assessed with a homemade Matlab program (The MathWorks, Inc.) by measuring the whole occupied cell area. This area is normalized by the initial area.

### Statistics

Values were compared using a non-parametric two-tailed Mann–Whitney test. The level of statistical significance was set at p<0.05. In figure captions, n represents the number of MCTS investigated for each particular condition or cell line.

## Results

### MCTS growth in 3D

**Fig 1:**
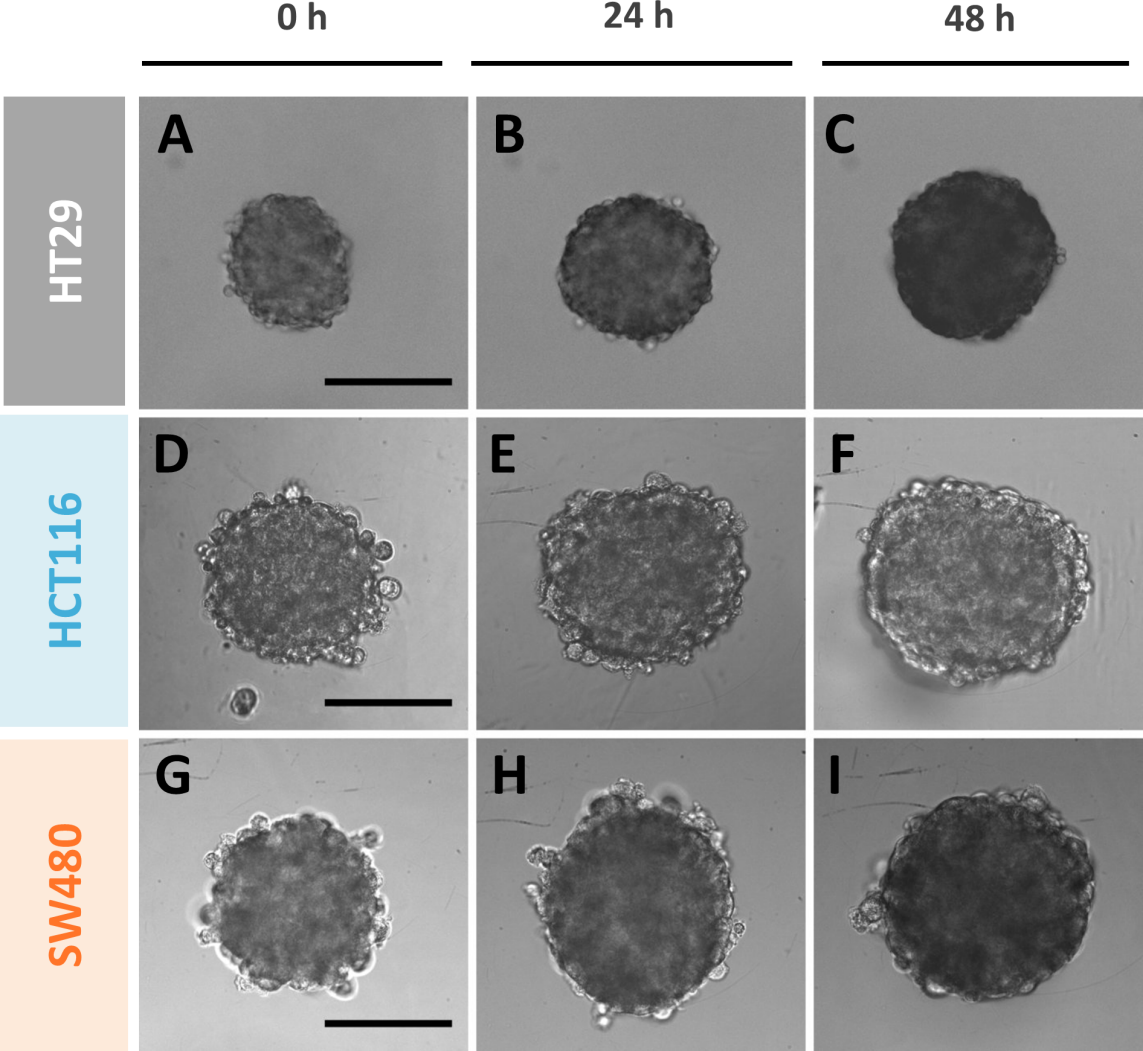
Typical phase contrast images of the MCTS growth for the three CRC lines taken at 0h, 24h and 48h in the absence of drug. **(A-C)** HT29, **(D-F)** HCT116 and **(G-I)** SW480. Scale bars: 200 μm.

Spheroid growth is assessed over time using classical bright field microscope. In the absence of drug, the MCTS mean diameter continuously increases with time from 219 ± 2 μm at day 0 to 277 ± 3 μm at day 2 (48h) for HT29 MCTS, 303 ± 3 μm to 358 ± 5 μm for HCT116 and 315 ± 3 μm to 364 ± 4 μm for SW480 (Fig 1). Assuming a spherical shape, the spheroid diameter or projected area extracted from optical microscopy images enables the calculation of the spheroid volume, which is a straightforward read-out of growth. The MCTS volume increases by a factor of two for HT29 cell line and slightly less for the two other cell lines.

### MCTS spreading on collagen to rank the cohesion degree of MCTS for the three CRC cell lines

Following the kinetics of spreading of MCTS placed on a surface coated with extracellular cell matrix (ECM) molecules is a simple assay to qualitatively characterize cell-cell cohesion [22], the slower the spreading the higher the cell-cell cohesion. Because, the difference between SW480 and HCT116 was not clearly reported in the literature, we performed this simple assay with our three CRC MCTS placed on collagen film. It appears that the three CRC cell lines tested do not invade inside the collagen (data not shown). By quantifying the spreading area, we show that HT29 are much more cohesive than the two other CRC cell lines. Among them, the HCT116 cell line is slightly but significantly more cohesive than the SW480 cell line (See supplementary **S1** Fig).

### An outer loose layer of cells breaks away from the MCTS under 5-FU treatment

**Fig 2:**
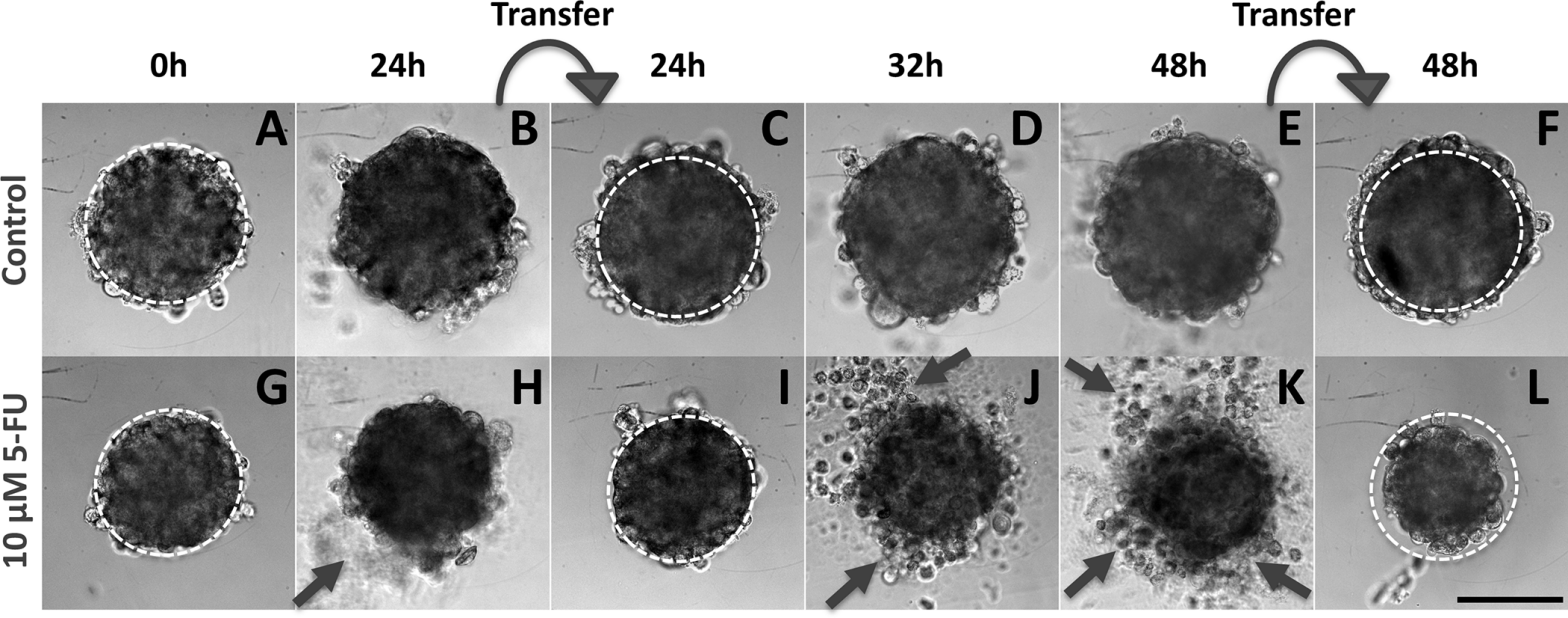
Typical images of the follow-up of a SW480 MCTS. Images taken at 0h, 24h, 32h and 48h in the absence of drug **(A-F)** or in presence of 10 μM of 5-FU dispensed at initial time 0h **(G-L)**. The dotted line representing the initial spheroid size in A, G is reported on other images as a guide for the eye. Curved arrows indicate the spheroids transfer into a new well. Straight short arrows show the diffuse cell outer layer. Scale bar: 200 μm.

**Fig 3:**
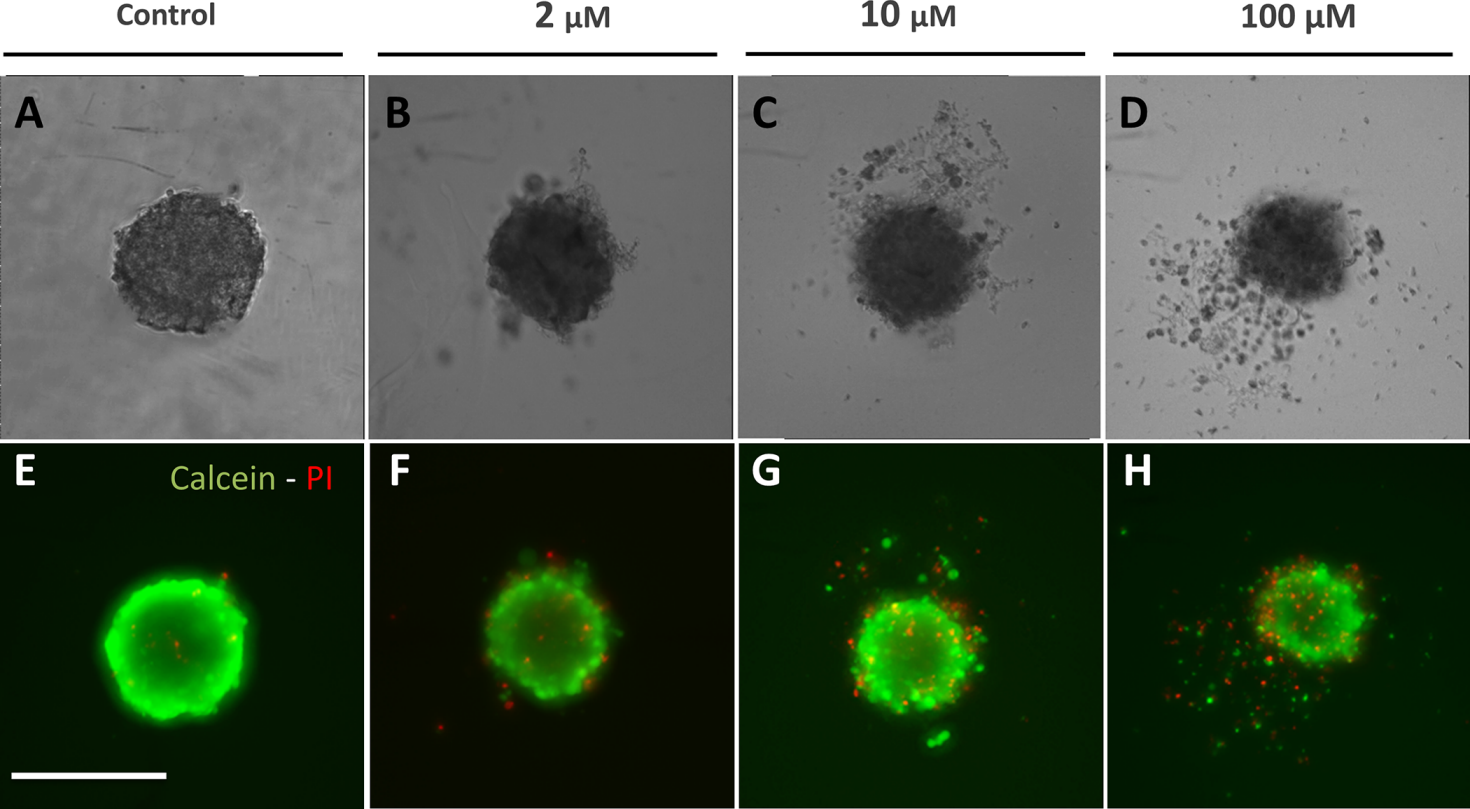
Nature of cells in the outer layerof the SW480 spheroids at 48h. **(A-D)** Phase contrast and **(E-H)** corresponding fluorescent images of living cells in green (calcein +) and dead cells in red (PI+) treated with 0, 2, 10 and 100 μM of 5-FU. Compared to control **(A,E)**, a gradual disaggregation of peripheral cells appears clearly since 2 μM of 5-FU **(B,F)** to 100 μM **(D,H)**. Cells in the outer layer are equally dead and living cells. Scale bar: 200 μm.

**Fig 4:**
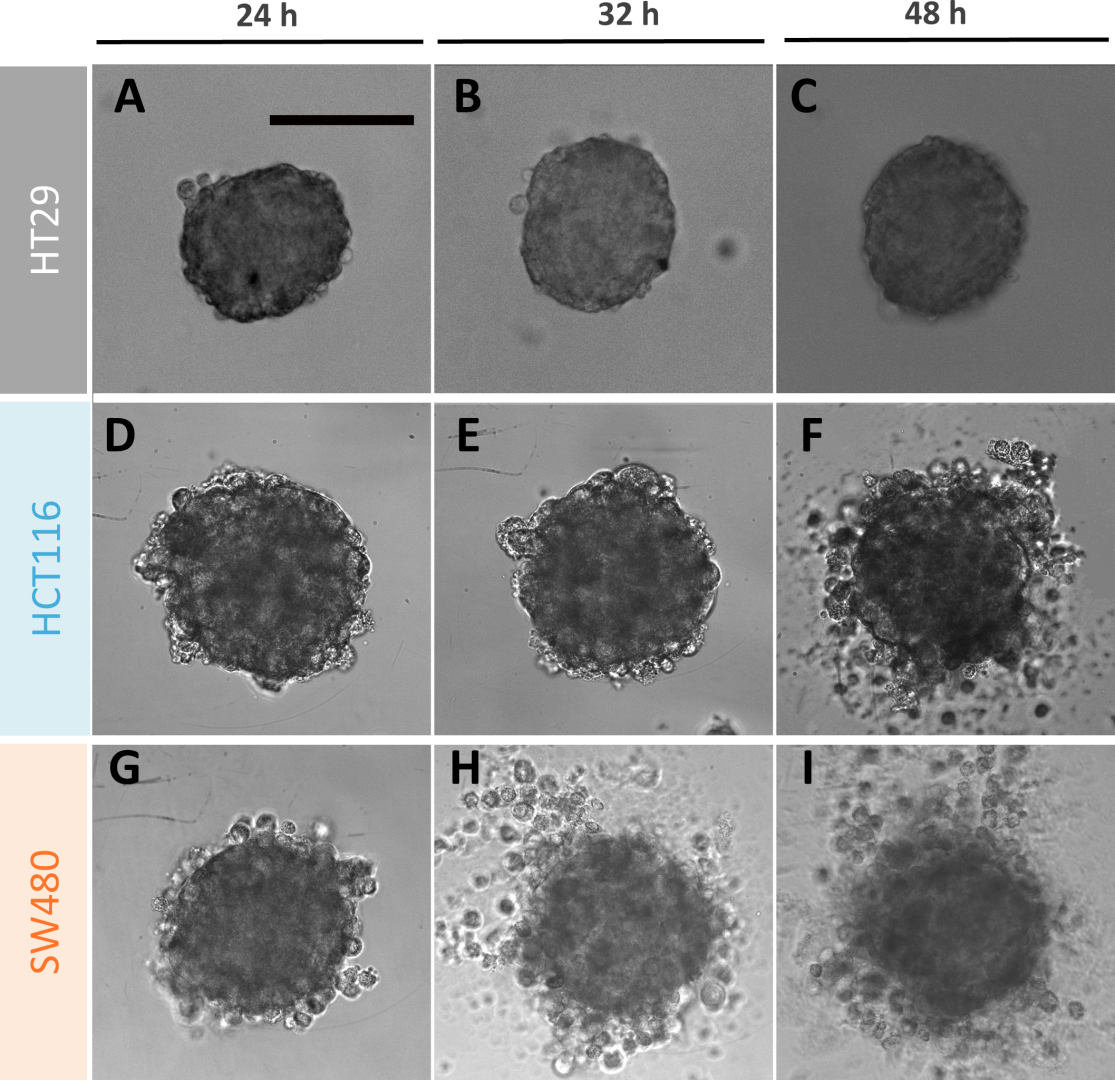
An outer loose layer of cells breaks away from the MCTS under 10 μM of 5-FU treatment. Typical images of MCTS of the three different CRC cell lines, HT29 (A-C), HCT116 (D-F) and SW480 (G-I) at three different times after drug administration ([5-FU] = 10μM): 24h (A, D, G), 32h (B, E, H) and 48h (C, F, I). In the absence of transfer, numerous dead cells appear on the diffuse outer cell layer for the HCT116 and SW480 MCTS. Scale bar: 200 μm.

When exposed to 5-FU, the measurement of the MCTS volume is not so straightforward as exemplified in Fig 2 with the case of the SW480 cell line (10 μM 5-FU condition). A diffuse outer layer of cells appears after 24h (Fig 2H). This layer is loosely attached to the core of the spheroid as a simple transfer by pipetting from one to another well with a fresh renewed medium with drug removes it (Fig 2I). Within eight supplementary hours (*i*.*e*., up to 32 h), loose cells against leave the MCTS core (Fig 2J) and at 48h we again performed a transfer to clean up the MCTS (Fig 2K–2L). We checked that control spheroids do not present this loose diffuse layer and that transferring them from one to another well does not change their shape and size (Fig 2A–2F). Using calcein and propidium iodide (PI) dyes (green and red respectively), we assessed at 48h the number of living and dead cells respectively as a function of the 5-FU concentration for SW480 cell line at day 2 without transfer (Fig 3). In order to keep most of the cells of the diffuse layer, we embedded gently the MCTS in agarose before staining cells (see materials and methods). Several important conclusions can be drawn from these observations. First, dead cells (PI+) are present both in the core and in the outer layer of the MCTS even if they are found at higher density (proportionnaly to the cellular volume) in the diffuse layer. Getting a closer look to this outer layer, we found that the number of dead cell is highly 5-FU concentration dependent (See supplementary S2 Fig). Finally and more surprisingly, the diffuse outer layer is composed not only of dead cells, but also of living cells, with a proportion of living or dead cell roughly equivalent in the outer layer whatever the 5-FU concentration (50%/50%, see supplementary S2 Fig). This last observation indicates that this outer layer is partially composed of live 5-FU resistant cells that detached from the core of the MCTS. For HCT116, we also observe the formation of an outer layer (Fig 4D–4F) but with a slight timing difference as it takes more than eight hours after the first transfer to rebuild it from scratch (Fig 4E versus Fig 4H for HCT116 and SW480 respectively, and quantification in supplementary S3 Fig). For HT29 MCTS, we do not observe any loose outer layer (Fig 4A–4C), the situation is similar to control MCTS.

### Death in the spheroid center

**Fig 5:**
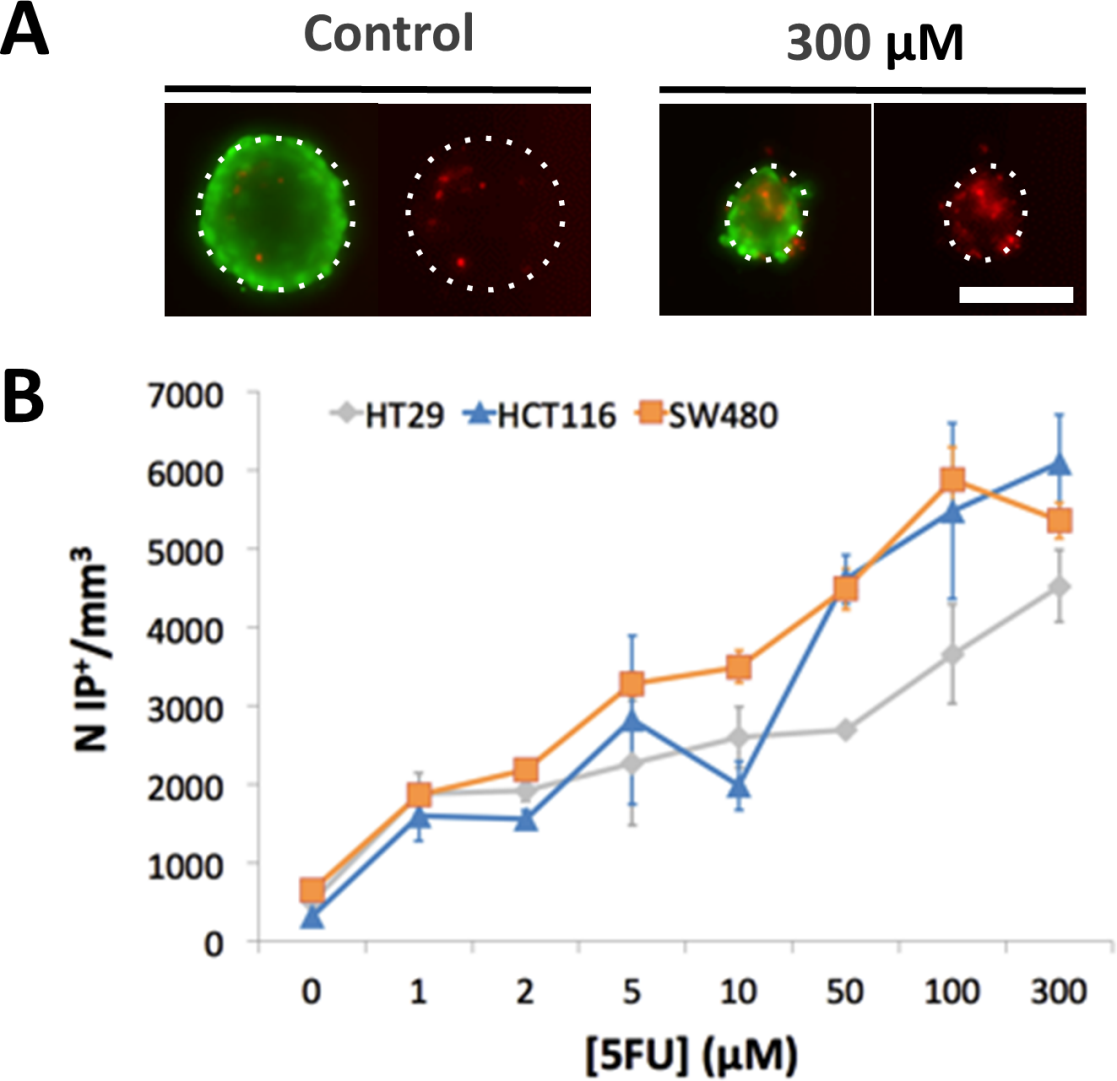
Number of dead cells within the cohesive core of MCTS under drug treatment after transfer to new wells. **(A)** Typical fluorescence images at 48h of living cells in green (calcein +) and dead cells in red (PI+) in control SW480 MCTS or in presence of 5-FU (300 μM). **(B)** Quantification of PI+ dead cells per unit volume for each cell line as a function of the 5-FU concentration. Error bar = SEM (standard error of the mean), (n = 12 for each cell line). Scale bar: 200 μm.

We computed the number of PI+ cells within the core of the MCTS at 48h after transferring the MCTS to a new plate in order to remove the diffuse layer. The number of PI+ particles increases proportionally to the concentration of 5-FU for all cell lines (Fig 5). A higher number of dead cells per unit volume is observed for SW480 spheroids at low 5-FU concentrations ([5–FU] = 5–10 μM) as compared to the other metastatic cell line HCT116 spheroids while HT29 spheroids present the lower number of PI+.

### Drug effect on the cohesive core growth

**Fig 6:**
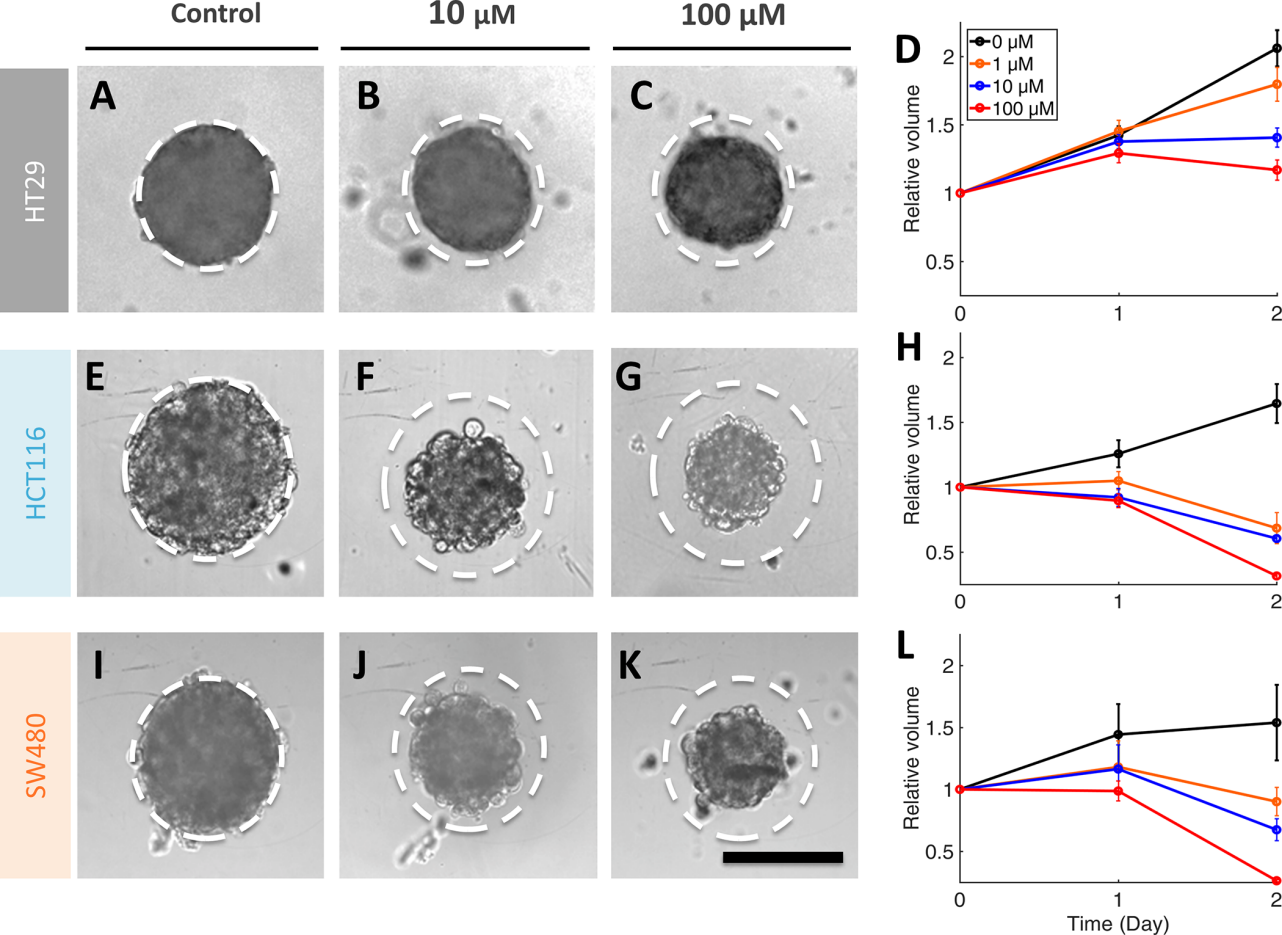
Volume of the cohesive core of MCTS under drug treatment after transfer to new wells. Left. Typical images of spheroid cores at 48h for the three CRC cell lines after administration of 0 **μM (A, E, I)**, 10 μM **(B, F, J)** or 100 μM **(C, G, K)** 5-FU. The dotted circle represents the size of the control MCTS which is reported as a guide for the eye on other panels. Right. Time series of the mean MCTS volume relative to initial volume at day 0 for each particular condition after 5-FU treatment ranging from 0 to 100 μM (only 3 drug concentrations are reported for clarity). **(A-D)** HT29, **(E-H)** HCT116 and **(I-L)** SW480. Error bars: SEM (n = 7–12 for each cell line). Scale bar: 200 μm.

**Fig 7:**
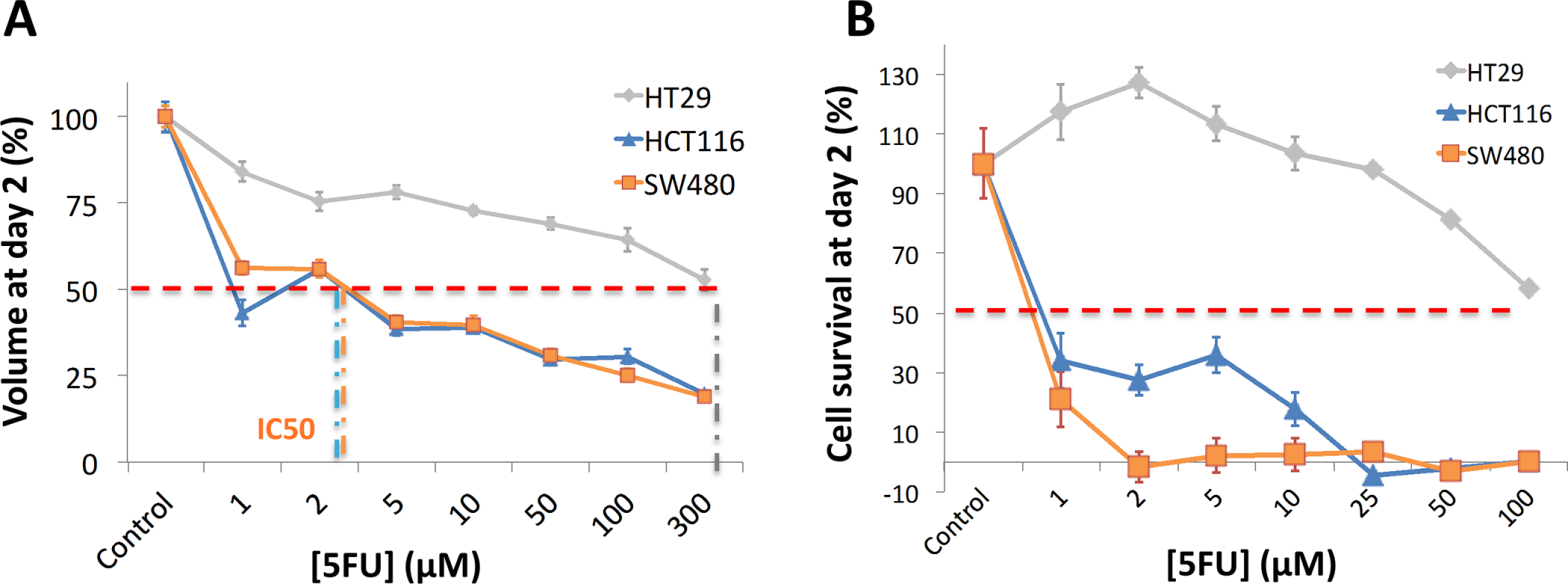
3D versus 2D 5-FU effect at 48h. **(A)** 3D cell survival of HCT116, SW480 and HT29 MCTS 48 hours after 5-FU treatment ranging from 0 to 100 μM. Survival represents the ratio of MCTS volume at 48h for a given drug condition to the volume of control MCTS (n = 7–12). **(B)** 2D cell survival of HCT116, SW480 and HT29 cell lines 48 hours after 5-FU treatment ranging from 0 to 100 μM. Survival represents the ratio of the normalized cell area at 48h for a given drug condition to the control normalized cell area (no drug) (n = 3). Error bars: SD.

The outer diffuse layer, if accounted in the area measurement, induces an overestimation of the growth. For that reason, we monitor every day MCTS growth after transferring the MCTS in a new well with fresh medium (Fig 2), leaving only the intact cohesive MCTS core but eliminating the peripheral loose cells (Fig 6). Quantitative measurements of the core volume are performed at 0h, 24h and 48h after transfer (Fig 6D, 6H and 6L). With respect to the control, the core volume gradually decreases as a function of the 5-FU concentration. 3D IC_50_ are estimated from the 50% value in 3D cell survival curves (Fig 7A at 48h). Here, 3D cell survival is defined as the ratio between the core volume for a given condition to the control volume at the same time. This quantity decreases continuously from 1 to 300 μM 5-FU. IC_50_ values at 24h and 48h are reported in Table 1: they are [1–5 μM] for both SW480 and HCT116 and larger than 300 μM for HT29. We clearly observe a higher resistance of HT29 spheroids against 5-FU in comparison with the higher metastatic ones HCT116 and SW480. In addition, as opposed to HCT116 and SW480, HT29 spheroids keep growing for all concentrations below 300 μM (Fig 6D) and are very cohesive even after two days’ drug exposure as judged by the absence of the diffuse cell layer (Fig 4).

**Table 1:** IC_50_ values for 5-FU for the three cell lines at 24h and 48h in 2D versus 3D spheroids. A range of IC_50_ values (e.g., 1–2 μM) indicates that the precise IC_50_ was in between two concentrations tested.

		24h			48h		
		SW480	HCT116	HT29	SW480	HCT116	HT29
IC_50_	2D	1-2 μM	50-100 μM	> 100 μM	< 1 μM	2-5 μM	100 μM
	3D	> 300 μM	> 300 μM	> 300 μM	1-5 μM	1-5 μM	> 300 μM

### Drug effect on 2D cell growth

**Fig 8:**
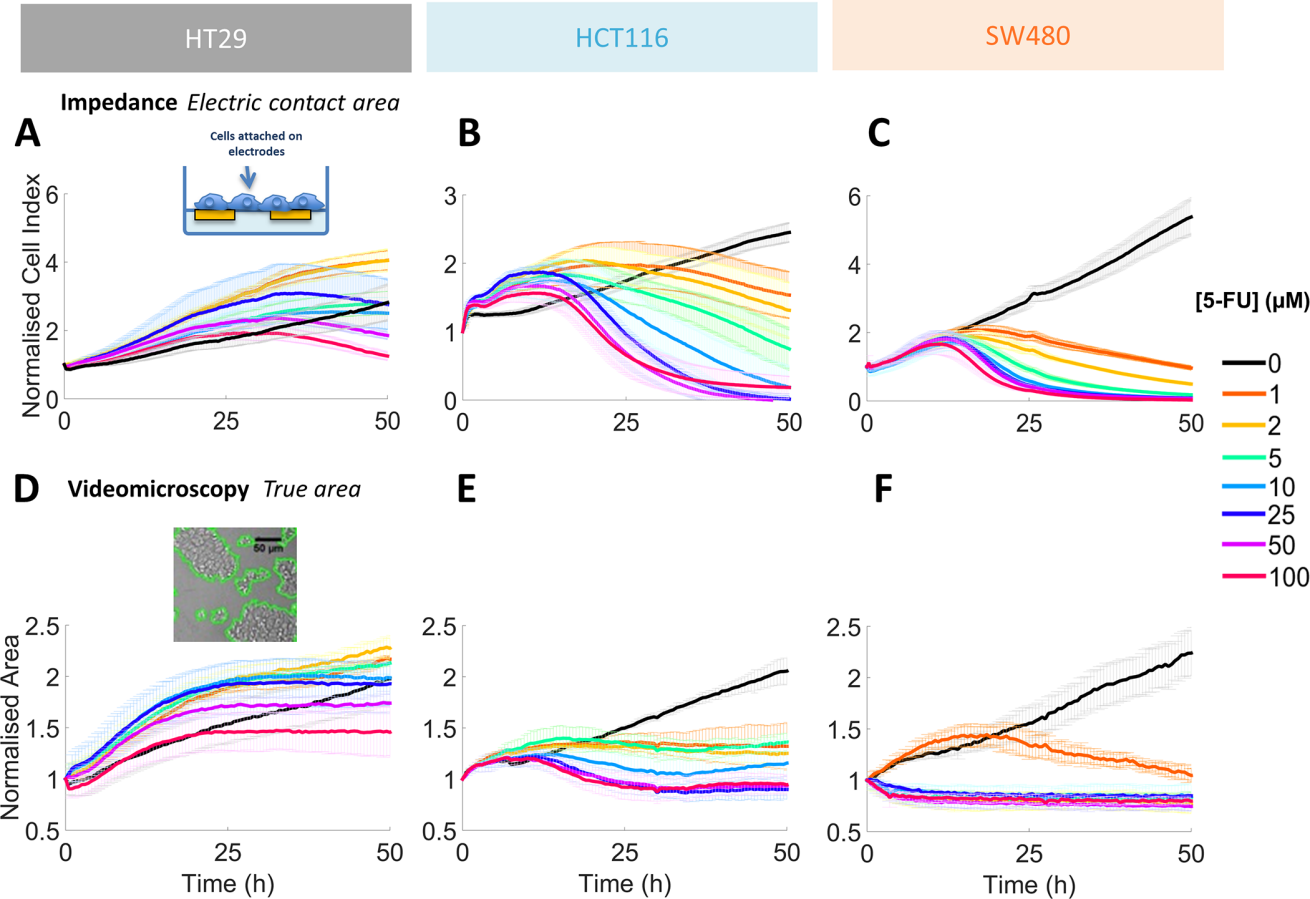
2D real time growth analysis. **(A-C)** Time series of the Cell Index obtained by RTCA impedance assay for the three CRC cell lines after 5-FU treatment administrated at initial time ([5FU] ranging from 0 to 100 μM, see colour code on the right). **(D-F)** Time series of the normalized cell area obtained by the videomicroscopy assay for the three CRC cell lines after 5-FU treatment administrated at initial time. Error bars: SD. Results from three distinct experiments in triplicate conditions.

In order to compare the drug effect obtained in 3D to the most classical one obtained in 2D, we also quantify the effect of 5-FU on 2-D cell growth of our three CRC cell lines using two complementary real time cell analysis methods, namely impedimetric and optical methods (Fig 8). The electrical signal collected via the impedimetric method (Cell Index) is correlated with the whole 2D cell area (Fig 8). This Cell Index is monotonously increasing over time for control conditions (no 5-FU added) for the three CRC cell lines. But it follows a non-monotonous cell line dependent variation in presence of the anti-cancer drug 5-FU. The Cell Index of the HT29 cell population is increasing faster than the control for all 5-FU concentrations. It saturates and cross the control curve at about 24h for 100 μM and at times larger than 48h for all other concentrations. The HCT116 Cell Index follows qualitatively the same trend but is more sensitive to the drug: it increases faster than the control at short times, it crosses the control curves for times comprised between 18h and 36h depending on the 5-FU concentration and finally decreases quickly to zero by 48h for concentrations larger than 10 μM. We will refer to cell activation in the following the fact that Cell Index (or area) during the first 18-48h after drug administration is larger than the control condition. In presence of drug, the SW480 Cell Index follows the control during the first 12h and drops in a concentration dependent manner abruptly by 24h for all concentrations larger than 5 μM.

To summarize, the response in term of Cell Index or whole cell area is highly cell line dependent. Cells are activated by the drug at short time-points, enter in a quiescent state at intermediate times and decrease at subsequent times due to cell death. Increasing the drug concentration fastens cell death. Except for HT29 with 5FU concentrations lower than 25 μM, the Cell Index with drug is always lower than the control at 48h. We computed the cell survival at 48h by computing the ratio of the area with drug to the control area at that time (Fig 7B). We next evaluated half maximal inhibitory concentration (IC_50_) values that indicate how much of 5-FU is needed to inhibit the cell proliferation by half (dotted line in Fig 7B). We also evaluated IC_50_ at 24h in case of 2D cell culture for comparison with 3D MCTS (Table 1).

### 3D versus 2D drug potency (IC_50_)

We have found in 2D the same hierarchy than in 3D, with both SW480 and HCT116 much more affected by the drug than HT29 MCTS. The comparison of IC_50_ values and cell survival curves at 48h indicates that MCTS are more resistant to 5-FU than 2D cultures. IC_50_ values are roughly 2 to 3 times larger in 3D than 2D for the three cell lines (Table 1). At 24 hours post 5-FU, we cannot determine any IC_50_ for HT29 both in 2D and 3D. But at that early time, interesting differences appear between the two metastatic cell lines. The IC_50_ for SW480 is 1–2 μM and 50–100 μM for HCT116 in 2D while it is not measurable in 3D due to a higher resistance.

As a summary, we found a higher resistance to the drug in 3D than in 2D especially at early times: it is necessary in 3D to wait longer times and administrate higher concentration for a same effect on cell survival. Among the two metastatic cell lines, the SW480 cell line is affected earlier by the drug, a phenomenon seen in 2D on IC_50_ values at 24h (IC_50_ of 2 μM for SW480 versus 50–100 μM for HCT116) and in 3D from the diffuse outer layer re-apparition at 32h (Fig 4, Supplementary S3 Fig).

## Discussion

This study investigated the response to 5-FU treatment of three CRC cell lines. While CRC cell sensitivity to 5-FU has been widely investigated using both 2D [23–26] and 3D models [27–31], to our best knowledge, none of them is comparing the response to 5-FU in terms of kinetics of toxicity and phenotypic analysis for different cell lines presenting different levels of invasive properties. In this study, we found different sensitivity to 5-FU for the three CRC cell lines, ranging from high (SW480), intermediate (HCT116) and low (HT29). We also observe the same hierarchy of CRC cell lines sensitivity between 3D and 2D two days after 5-FU exposure. Moreover, for the three studied cell lines, the 2D IC_50_ values are two to three times smaller than in 3D at 48h. Our estimates are comparable to the literature reporting a higher resistance to 5-FU in 3D CRC cell models [13,27–32] and a higher sensitivity for HCT116 compare to HT29 cell lines both in 2D [23–26] and in 3D [7]. As far as the SW480 cell line is concerned, the 5-FU response has never been reported to our best knowledge, despite its relevance as a model of CRC progression [33].

While in 2D the cell survival at 48h is close to zero at a concentration above 5 μM (for SW480) and 25 μM (for HCT116), we show that a core full of living cells is still present in all MCTS at that time, representing more than 25% of the control core volume. The presence of these living cells may be attributed to (1) a decrease in drug penetration in the center of the spheroid and/or (2) the presence of cells showing an increase resistance to 5-FU treatment in a 3D environment. Numerous studies in various tumoral cell lines have described a good penetration of 5-FU into spheroids compared to other drugs [34–37]. Cell resistance to 5-FU treatment may thus rather be related to a difference in cell-cell and cell-matrix interactions within the spheroid, compared to cells in 2D cultures.

### Spheroid cohesion as an indicator of cell sensitivity to 5-FU

It was reported that tissue cohesion influences the resistance to drugs treatment for HT29 [38]. Several lines of argument indicate that HT29 is much more cohesive than the two metastatic cell lines [39]. HT29 cells are rich in cadherin/catenin cell-to-cell junctions providing a cohesive character implicated in 3D drug resistance [38]. Cadherins are classical cell–cell adhesion molecules and their down-regulation is often associated with increased invasive potential in tumors of epithelial origins [40]. SW480 cells, when confluent, grew in 2D as less cohesive cell clusters, showing only partial membranous localization of E-cadherin and catenins at sites of cell–cell contacts. The membranous localization of E-cadherin and catenins is much stronger for HCT116 and even more for HT29 [39]. To confirm the cohesion degree of our cell lines, we used the method of sequential spheroid trypsinization to obtain qualitatively an index of cell cohesion described before by Carlsson and Nederman [41]. As expected, HT29 MCTS are much more difficult to disrupt than the two other cell types, reflecting their high degree of cohesion. Moreover, increasing cell–cell cohesion decreases spheroid spreading rate on a given substratum [22]. Using such a simple assay of spheroid spreading on a collagen type I, we showed that HT29 cells spread much slower than the two other cell lines HCT116 and SW480 with high spreading potential (S1 Fig). These features are correlated with the cohesion degree of the three cell lines previously described.

It has been shown that adhesion molecules including cadherins, integrins and various components of the ECM can alter tumor mechanical properties [42,43], and hence, cohesion. Cells with an extensive ECM are more susceptible to grow as multicellular spheroids and less subject to disintegration [44]. Moreover, laminin and collagen I, two major components of the ECM and present in large quantities in HT29 spheroids have been reported to be implicated in cell-matrix signaling as a major regulator of various functions of cancer cells [30]. Many studies are now pointing out the role of mechanical cues in tumour progression, and consequently the modification of cell mechanical properties during the metastatic process (reviewed recently in [45], and more specifically for HT29 and SW480 in [46]). In this line, we evidence in this study the presence of a detaching ring of cells for the less cohesive cell lines, allowing us to speculate on an anti-cohesive character to the 5-FU drug treatment, probably due to cell-cell or cell-matrix adhesion properties.

### A novel simple method to quantify cell cohesion and drug resistance

We want to stress out that most of the studies dealing with 3D spheroids are quantifying cell sensitivity to drugs by simply monitoring the volume growth over time, extracted from phase contrast images without considering any gradual change in MCTS density such as the presence of an outer diffuse layer [30]. This approach is working very well in case of a cohesive cancer cell line such as HT29 (grade I) [47]. However, if one wants to focus on drug sensitivity for cells exhibiting higher grades of cancer or for primary cells, the drugs could induce a rapid loss of spheroid cohesion, *i*.*e*., a quick disaggregation of the outer cells layer. This was the case in our study for HCT116 and SW480 (both grade IV). Here, the follow-up of the projected area can lead to completely opposite results. In the present study, by transferring the spheroids at regular time point, we have presented a simple yet robust method to disentangle drug effect on cell proliferation from the effect on cell disaggregation. Note that not only the remaining core, but also some detaching cells are viable cells. These few detaching cells remaining fully active (alive and still capable to spread on collagen type I) are of particular interest as it represents the most resistant subpopulation that should be eliminated to avoid any relapse.

### 5-FU response: 2D cell model reveals cell activation during the first hours of treatment

Surprisingly, we observed an overstimulation of cell growth in 2D the firsts 24 hours after 5-FU mainly for low doses. This interesting effect has been reported recently [26] and can be associated to a mitotic stress after drug addition [23, 48]. It should be pointed out that it is much more difficult to evidence such a dynamic effect in 3D cell model. Indeed, to decipher such increase in mitotic activity and/or mean cell volume within spheroids would require the use of specific optical techniques such as Light Sheet Microscopy or labour intensive immunostaining. As such, this study shows that 2D and 3D cell culture models bring complementary results. 2D models are easy to handle, and even if (1) cells are far from the *in vivo* conditions and (2) the drug dose response is shifted with respect to 3D models, the hierarchy between the resistance of various cell lines to 5-FU is conserved. More importantly, they give information on cellular dynamic that would be difficult to evidence in 3D.

## Conclusions

The identification of active cancer drugs or the combined use of drugs whose action spectrum affects both cohesive and invasive cells is fundamental to increase chemotherapy efficacy [40,49,50]. The proposed methodology yet simple could be a tool readily accessible to analyze the effect of various drugs on spheroids cohesion, a model closer to *in vivo* conditions that traditional 2D cell culture systems. The use of this methodology for CRC cell lines confirms the high resistance to 5-FU treatment of the cohesive HT29 cell line and highlights subtle differences in the sensitivity to 5-FU treatment between the two invasive cell lines SW480 and HCT116. Interestingly, these differences are fully correlated with the MCTS cohesion properties. The early activation that we quantified for HT29 and HCT116 cell lines is very surprising, and we are currently analyzing this effect in more detail. The quantification of the internal structure of spheroids could enable to decipher more precisely the phenotypic changes of cells within the spheroid under 5-FU treatment.

## Supporting information

S1 Fig Spreading of the three CRC spheroids on a collagen type I film. (A-I) Typical images of MCTS taken with the transmission channel of the confocal for the three cell lines at initial time 0h (A, D, G), 24h (B,E,H) and 48h (C,F,I) after depositing the spheroid on the collagen film. The culture medium was not renewed and does not contain 5-FU. Scale bar, 500 μm. (J) Kinetics of spreading (normalized projected cell area Vs. time after deposition). The slower the spreading, the higher is the cell-cell cohesion. Error bars represent SEM (n = 3–5 for each cell line).
(TIF)

S2 Fig Quantification of live (calcein) and dead (PI) cells in the outer cells layer of SW480 spheroids treated with 0, 2, 10 and 100 μM of 5-FU at day 2. Left: the disaggregated outer layer is composed of both live and dead cells (50–50%) at all 5-FU concentrations. Right: the number of dead cells in the outer layer increases gradually with the 5-FU concentration. Because of the difficulty to define the exact boundary between the MCTS core and the outer layer and because some peripheral cells might be lost during the agarose injection step all around the spheroid (see materials and methods), it is difficult to compare quantitatively the number of cells inside and outside the MCTS core. On the other hand, the transfer technique enables a precise quantification of the number of dead cells in the MCTS core (See Fig 5). Error bars: SEM.
(TIF)

S3 Fig Spheroid relative diameter change between 24h (after transfer) and 32h for the two invasive CRC cell lines. (A) Relative diameter change as a function of the 5-FU concentration. The diameter is evaluated from the spheroid surface area A including the diffuse outer layer measurement as (4A/π^1/2^. Error bars represent SEM (n = 7–12 for each cell line). (B,E) Typical images of MCTS at 24h (after transfer) and 32h for 10μM 5-FU. Scale bar, 200 μm.
(TIF)

## References

[1] Kalashnikova I, Albekairi N, Ali S, Al Enazy S and Rytting E. Cell Culture Models for Drug Transport Studies, in Drug Delivery: Principles and Applications, Second Edition (eds Wang B., Hu L. and Siahaan T. J.), John Wiley & Sons, Inc, Hoboken, NJ 2016 doi: 10.1002/9781118833322.ch7

[2] Wirtz D, Konstantopoulos K, Searson PC. The physics of cancer: the role of physical interactions and mechanical forces in metastasis. Nat Rev Cancer. 2011;11: 512–522. doi: 10.1038/nrc3080 21701513 10.1038/nrc3080 PMC3262453

[3] Pickup MW, Mouw JK, Weaver VM. The extracellular matrix modulates the hallmarks of cancer. EMBO Rep. 2014;15:1243–53. doi: 10.15252/embr.201439246 25381661 10.15252/embr.201439246 PMC4264927

[4] Mehta G, Hsiao AY, Ingram M, Luker GD, Takayama S. Opportunities and challenges for use of tumor spheroids as models to test drug delivery and efficacy. J Control Release. 2012;164: 192–204. doi: 10.1016/j.jconrel.2012.04.045 22613880 10.1016/j.jconrel.2012.04.045 PMC3436947

[5] Sutherland RM, Inch WR, McCredie JA, Kruuv J. A multi-component radiation survival curve using an in vitro tumour model. Int J Radiat Biol Relat Stud Phys Chem Med. 1970;18: 491–495 5316564 10.1080/09553007014551401

[6] Sutherland RM, McCredie JA, Inch WR. Growth of multicell spheroids in tissue culture as a model of nodular carcinomas. J Natl Cancer Inst. 1971;46: 113–120 5101993

[7] Friedrich J, Ebner R, Kunz-Schughart LA. Experimental anti-tumor therapy in 3-D: Spheroids–old hat or new challenge? Int J Radiat Biol. 2007;83: 849–871. doi: 10.1080/09553000701727531 18058370 10.1080/09553000701727531

[8] Friedrich J, Eder W, Castaneda J, Doss M, Huber E, Ebner R, et al A reliable tool to determine cell viability in complex 3-d culture: the acid phosphatase assay. J Biomol Screen. 2007;12: 925–37. doi: 10.1177/1087057107306839 17942785 10.1177/1087057107306839

[9] Senkowski W, Zhang X, Olofsson MH, Isacson R, Höglund U, Gustafsson M, et al Three-Dimensional Cell Culture-Based Screening Identifies the Anthelmintic Drug Nitazoxanide as a Candidate for Treatment of Colorectal Cancer. Mol Cancer Ther. 2015;14: 1504–16. doi: 10.1158/1535-7163.MCT-14-0792 25911689 10.1158/1535-7163.MCT-14-0792

[10] Katt ME, Placone AL, Wong AD, Xu ZS, Searson PC. In Vitro Tumor Models: Advantages, Disadvantages, Variables, and Selecting the Right Platform. Front Bioeng Biotechnol. 2016;4 doi: 10.3389/fbioe.2016.00012 26904541 10.3389/fbioe.2016.00012 PMC4751256

[11] Laurent J, Frongia C, Cazales M, Mondesert O, Ducommun B, Lobjois V. Multicellular tumor spheroid models to explore cell cycle checkpoints in 3D. BMC cancer. 2013 p. 73 doi: 10.1186/1471-2407-13-73 23394599 10.1186/1471-2407-13-73 PMC3598667

[12] Kunz-Schughart LA & Freyer JP. Adaptation of an automated selective dissociation procedure to two novel spheroid types. In Vitro Cell Dev Biol Anim. 1997;33(2), 73–6 9081211 10.1007/s11626-997-0024-3

[13] Shi W Bin Le VM, Gu CH Zheng YH, Lang MD Lu YH, et al Overcoming multidrug resistance in 2D and 3D culture models by controlled drug chitosan-graft poly(caprolactone)-based nanoparticles. J Pharm Sci. Elsevier Masson SAS; 2014;103: 1064–1074. doi: 10.1002/jps.23860 24523221 10.1002/jps.23860

[14] Focaccetti C, Bruno A, Magnani E, Bartolini D, Principi E, Dallaglio K, et al Effects of 5-fluorouracil on morphology, cell cycle, proliferation, apoptosis, autophagy and ros production in endothelial cells and cardiomyocytes. PLoS One. 2015;10: 1–25. doi: 10.1371/journal.pone.0115686 25671635 10.1371/journal.pone.0115686 PMC4324934

[15] Friedrich J, Seidel C, Ebner R, Kunz-Schughart LA. Spheroid-based drug screen: considerations and practical approach. Nat Protoc. 2009;4: 309–324. doi: 10.1038/nprot.2008.226 19214182 10.1038/nprot.2008.226

[16] Adcock AF, Trivedi G, Edmondson R, Yang CS and L. Three-Dimensional (3D) Cell Cultures in Cell-based Assays for in-vitro Evaluation of Anticancer Drugs. J Anal Bioanal Tech. 2015;6 doi: 10.4172/2155-9872.1000249

[17] Yue X, Lukowski JK, Weaver EM, Skube SB, Hummon AB. Quantitative Proteomic and Phosphoproteomic Comparison of 2D and 3D Colon Cancer Cell Culture Models. J Proteome Res. 2016;15: 4265–4276. doi: 10.1021/acs.jproteome.6b00342 27696853 10.1021/acs.jproteome.6b00342 PMC5334570

[18] Qureshi-Baig K, Ullmann P, Rodriguez F, Frasquilho S, Nazarov P V., Haan S, et al What do we learn from spheroid culture systems? Insights from tumorspheres derived from primary colon cancer tissue. PLoS One. 2016;11 doi: 10.1371/journal.pone.0146052 26745821 10.1371/journal.pone.0146052 PMC4706382

[19] Luca AC, Mersch S, Deenen R, Schmidt S, Messner I, Schäfer KL, et al Impact of the 3D Microenvironment on Phenotype, Gene Expression, and EGFR Inhibition of Colorectal Cancer Cell Lines. PLoS One. 2013;8 doi: 10.1371/journal.pone.0059689 23555746 10.1371/journal.pone.0059689 PMC3608563

[20] Santo V. E., Rebelo S. P., Estrada M. F., Alves P. M., Boghaert E., & Brito C. (2017). Drug screening in 3D in vitro tumor models: overcoming current pitfalls of efficacy read-outs. Biotechnology Journal, 12(1), 1600505 http://doi.org/10.1002/biot.201600505 10.1002/biot.201600505 27966285

[21] Longley DB, Harkin DP, Johnston PG. 5-Fluorouracil: Mechanisms of Action and Clinical Strategies. Nat Rev Cancer. 2003;3: 330–338. doi: 10.1038/nrc1074 12724731 10.1038/nrc1074

[22] Ryan PL, Foty RA, Kohn J, Steinberg MS. Tissue spreading on implantable substrates is a competitive outcome of cell-cell vs. cell-substratum adhesivity. Proc Natl Acad Sci. 2001;98: 4323–4327. doi: 10.1073/pnas.071615398 11274361 10.1073/pnas.071615398 PMC31833

[23] De Angelis PM, Svendsrud DH, Kravik KL, Stokke T. Cellular response to 5-fluorouracil (5-FU) in 5-FU-resistant colon cancer cell lines during treatment and recovery. Mol Cancer. 2006;5: 20 doi: 10.1186/1476-4598-5-20 16709241 10.1186/1476-4598-5-20 PMC1524802

[24] Mhaidat NM, Bouklihacene M, Thorne RF. 5-Fluorouracil-induced apoptosis in colorectal cancer cells is caspase-9-dependent and mediated by activation of protein kinase C-δ. Oncol Lett. 2014;8: 699–704. doi: 10.3892/ol.2014.2211 25013487 10.3892/ol.2014.2211 PMC4081407

[25] Arul M, Roslani AC, Cheah SH. Heterogeneity in cancer cells: variation in drug response in different primary and secondary colorectal cancer cell lines in vitro. Vitr Cell Dev Biol—Anim. In Vitro Cellular & Developmental Biology—Animal; 2017; doi: 10.1007/s11626-016-0126 10.1007/s11626-016-0126-x 28120247

[26] Bash-Imam Z, Thérizols G, Vincent A, Lafôrets F, Polay Espinoza M, Pion N, Macari F, Pannequin J, David A, Saurin JC, Mertani HC, Textoris J, Auboeuf D, Catez F, Dalla Venezia N, Dutertre M, Marcel V, Diaz JJ. Translational reprogramming of colorectal cancer cells induced by 5-Fluorouracil through a miRNA-dependent mechanism. Oncotarget. 2017 5 3 doi: 10.18632/oncotarget.17597 28515355 10.18632/oncotarget.17597 PMC5542262

[27] Karlsson H, Fryknäs M, Larsson R, Nygren P. Loss of cancer drug activity in colon cancer HCT-116 cells during spheroid formation in a new 3-D spheroid cell culture system. Exp Cell Res. Elsevier Inc.; 2012;318: 1577–1585. doi: 10.1016/j.yexcr.2012.03.026 22487097 10.1016/j.yexcr.2012.03.026

[28] Charoen KM, Fallica B, Colson YL, Zaman MH, Grinstaff MW. Embedded multicellular spheroids as a biomimetic 3D cancer model for evaluating drug and drug-device combinations. Biomaterials. Elsevier Ltd; 2014;35: 2264–2271. doi: 10.1016/j.biomaterials.2013.11.038 24360576 10.1016/j.biomaterials.2013.11.038 PMC3923358

[29] Hoffmann OI, Ilmberger C, Magosch S, Joka M, Jauch KW, Mayer B. Impact of the spheroid model complexity on drug response. J Biotechnol. Elsevier B.V.; 2015;205: 14–23. doi: 10.1016/j.jbiotec.2015.02.029 25746901 10.1016/j.jbiotec.2015.02.029

[30] Thakuri PS, Ham SL, Luker GD, Tavana H. Multiparametric Analysis of Oncology Drug Screening with Aqueous Two-Phase Tumor Spheroids. Mol Pharm. 2016;13: 3724–3735. doi: 10.1021/acs.molpharmaceut.6b00527 27653969 10.1021/acs.molpharmaceut.6b00527

[31] Sagara A, Igarashi K, Otsuka M, Karasawa T, Gotoh N, Narita M, et al Intrinsic Resistance to 5-Fluorouracil in a Brain Metastatic Variant of Human Breast Cancer Cell Line, MDA-MB-231BR. PLoS One. 2016;11: e0164250 doi: 10.1371/journal.pone.0164250 27723829 10.1371/journal.pone.0164250 PMC5056764

[32] Fan X, Ouyang N, Teng H, Yao H. Isolation and characterization of spheroid cells from the HT29 colon cancer cell line. Int J Colorectal Dis. 2011;26: 1279–1285. doi: 10.1007/s00384-011-1248-y 21670985 10.1007/s00384-011-1248-y

[33] Hewitt RE, McMarlin A, Kleiner D, Wersto R, Martin P, Tsokos M, et al Validation of a model of colon cancer progression. J Pathol. 2000;192: 446–54. doi: 10.1002/1096-9896(2000)9999:9999<::AID-PATH775>3.0.CO;2-K 11113861 10.1002/1096-9896(2000)9999:9999<::AID-PATH775>3.0.CO;2-K

[34] Nederman T, Carlsson J. Penetration and binding of vinblastine and 5-fluorouracil in cellular spheroids. Cancer Chemother Pharmacol. 1984;13: 131–135. doi: 10.1007/BF00257130 6467498 10.1007/BF00257130

[35] Erlichman C, Wu A. Effects of 5-fluorouracil and leucovorin in spheroids: A model for solid tumours. Anticancer Res. 1991;11: 671–675 2064321

[36] Lowthers EL, Richard CL, Blay J. Differential sensitivity to short-chain ceramide analogues of humans intestinal carcinoma cells grown in tumor spheroids versus monolayer culture. In Vivo Cellular & Developmental Biology–Animal. 2003:39(8), 340 doi: 10.1290/1543-706X(2003)039<0340:DSTSCA>2.0.CO;2 10.1290/1543-706X(2003)039<0340:DSTSCA>2.0.CO;2 14640787

[37] Choi MS, Kim SH, Kuh HJ. Penetration of paclitaxel and 5-fluorouracil in multicellular layers of human colorectal cancer cells. Oncol Rep. 2011;25: 863–870. doi: 10.3892/or.2011.1138 21225235 10.3892/or.2011.1138

[38] Green SK, Francia G, Isidoro C, Kerbel RS. Antiadhesive antibodies targeting E-cadherin sensitize multicellular tumor spheroids to chemotherapy in vitro. Mol Cancer Ther. 2004;3: 149–159 14985455

[39] El-Bahrawy M, Poulsom SR, Rowan AJ, Tomlinson IT, Alison MR. Characterization of the E-cadherin/catenin complex in colorectal carcinoma cell lines. Int J Exp Pathol. 2004;85: 65–74. doi: 10.1111/j.0959-9673.2004.0371.x 15154912 10.1111/j.0959-9673.2004.0371.x PMC2517458

[40] Foty RA. Tumor cohesion and glioblastoma cell dispersal. Future Oncol. 2013;9: 1121–32. doi: 10.2217/fon.13.66 23902244 10.2217/fon.13.66 PMC3881193

[41] Carlsson J, Nedermant T. Tumour spheroid technology in cancer therapy research. Eur J Cancer Clin Oncol. 1989;25,1127–33 2670583 10.1016/0277-5379(89)90404-5

[42] Grantab R, Sivananthan S, Tannock IF. The penetration of anticancer drugs through tumor tissue as a function of cellular adhesion and packing density of tumor cells. Cancer Res. 2006;66: 1033–1039. doi: 10.1158/0008-5472.CAN-05-3077 16424039 10.1158/0008-5472.CAN-05-3077

[43] Spill F, Reynolds DS, Kamm RD, Zaman MH. Impact of the physical microenvironment on tumor progression and metastasis. Curr Opin Biotechnol. 2016;40 doi: 10.1016/j.copbio.2016.02.007 26938687 10.1016/j.copbio.2016.02.007 PMC4975620

[44] Glimelius B, Norling B, Nederman T, Carlsson J. Extracellular matrices in multicellular spheroids of human glioma origin: increased incorporation of proteoglycans and fibronectin as compared to monolayer cultures. Apmis. 1988;96: 433–444 3288248 10.1111/j.1699-0463.1988.tb05327.x

[45] Ciasca G., Papi M., Minelli E., Palmieri V., & De Spirito M. Changes in cellular mechanical properties during onset or progression of colorectal cancer. World Journal of Gastroenterology, 2016: 22(32), 7203–14. doi: 10.3748/wjg.v22.i32.7203 27621568 10.3748/wjg.v22.i32.7203 PMC4997642

[46] Pachenari M, Seyedpour SM, Janmaleki M, Shayan SB, Taranejoo S, Hosseinkhani H. Mechanical properties of cancer cytoskeleton depend on actin filaments to microtubules content: Investigating different grades of colon cancer cell lines. J Biomech. 2014;47: 373–379. doi: 10.1016/j.jbiomech.2013.11.020 24315289 10.1016/j.jbiomech.2013.11.020

[47] Däster S, Amatruda N, Calabrese D, Ivanek R, Turrini E, Droeser RA, et al Induction of hypoxia and necrosis in multicellular tumor spheroids is associated with resistance to chemotherapy treatment. Oncotarget. 2016;8: 1725–1736. doi: 10.18632/oncotarget.13857 27965457 10.18632/oncotarget.13857 PMC5352092

[48] Deng YC, Zhen YS, Zheng S, Xue YC. Activity of boanmycin against colorectal cancer. World J Gastroenterol. 2001;7: 93–97 doi: 10.3748/wjg.v7.i1.93 11819740 10.3748/wjg.v7.i1.93 PMC4688709

[49] Schmidmaier R, Baumann P. Anti-adhesion evolves to a promising therapeutic concept in oncology. Curr Med Chem. 2008;15: 978–990. doi: 10.2174/092986708784049667 18393855 10.2174/092986708784049667

[50] Lu CL, Xu J, Yao HJ, Luo KL, Li JM, Wu T, et al Inhibition of human 67-kDa laminin receptor sensitizes multidrug resistance colon cancer cell line SW480 for apoptosis induction. Tumor Biol. 2016;37: 1319–1325. doi: 10.1007/s13277-015-3873-5 26293895 10.1007/s13277-015-3873-5

