# Supplementary figures and images for: In-depth phenotypic characterization of multicellular tumor spheroids: Effects of 5-Fluorouracil
Source: PLoS One. 2017 Nov 15;12(11):e0188100. doi: 10.1371/journal.pone.0188100 (PMC5687732; doi:10.1371/journal.pone.0188100)

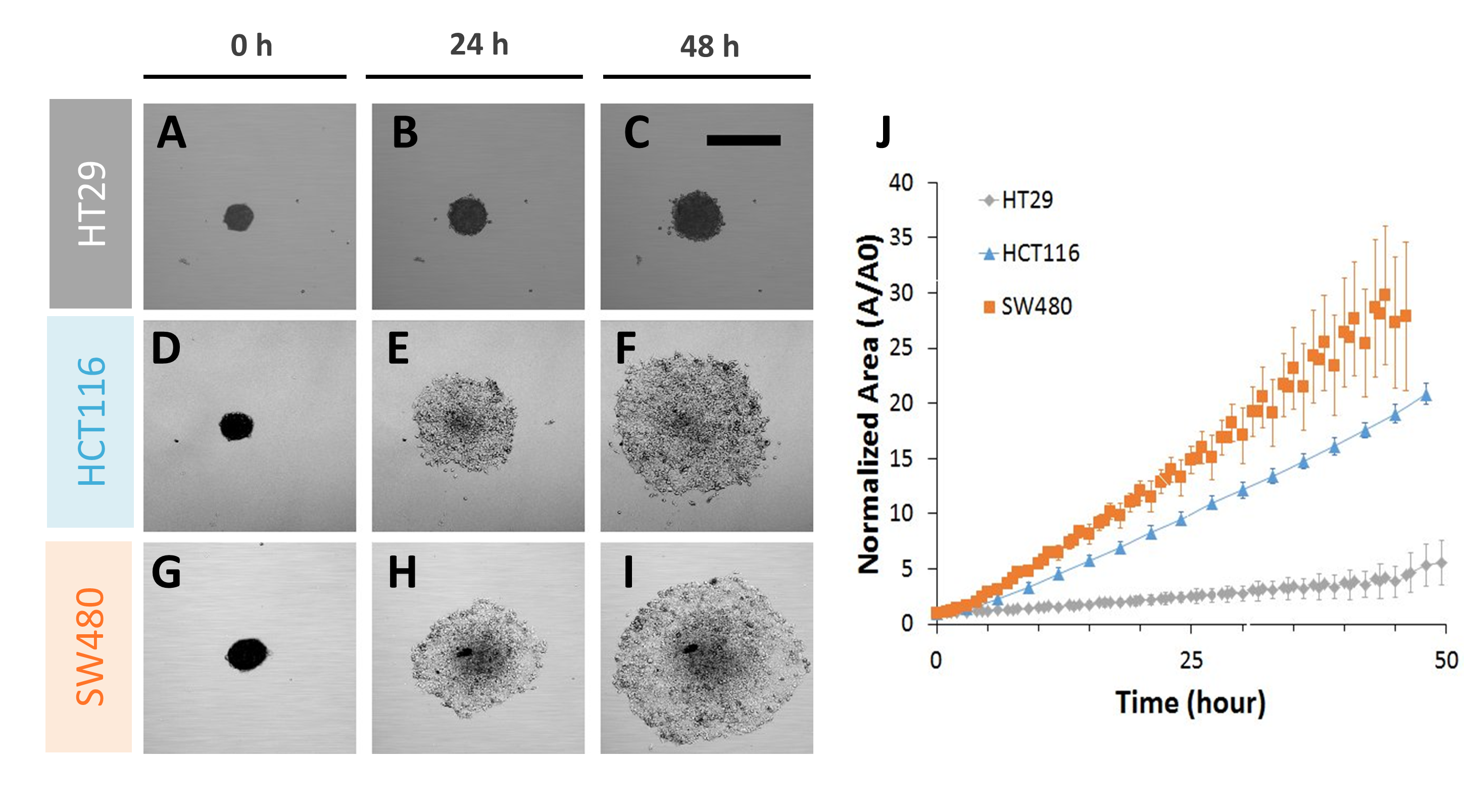

Supplement: S1 Fig — (A-I) Typical images of MCTS taken with the transmission channel of the confocal for the three cell lines at initial time 0h (A, D, G), 24h (B,E,H) and 48h (C,F,I) after depositing the spheroid on the collagen film. The culture medium was not renewed and does not contain 5-FU. Scale bar, 500 μm. (J) Kinetics of spreading (normalized projected cell area Vs. time after deposition). The slower the spreading, the higher is the cell-cell cohesion. Error bars represent SEM (n = 3–5 for each cell line). (TIF) [file pone.0188100.s001.tif]

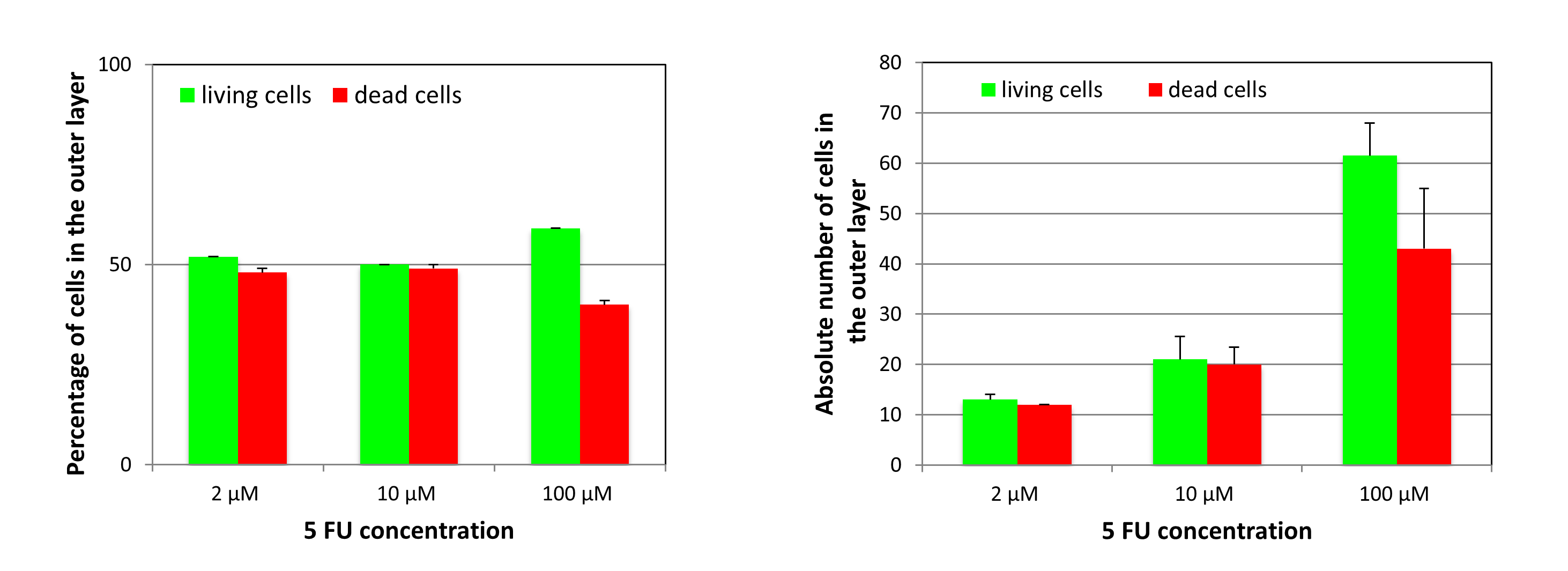

Supplement: S2 Fig — Left: the disaggregated outer layer is composed of both live and dead cells (50–50%) at all 5-FU concentrations. Right: the number of dead cells in the outer layer increases gradually with the 5-FU concentration. Because of the difficulty to define the exact boundary between the MCTS core and the outer layer and because some peripheral cells might be lost during the agarose injection step all around the spheroid (see materials and methods), it is difficult to compare quantitatively the number of cells inside and outside the MCTS core. On the other hand, the transfer technique enables a precise quantification of the number of dead cells in the MCTS core (See Fig 5). Error bars: SEM. (TIF) [file pone.0188100.s002.tif]

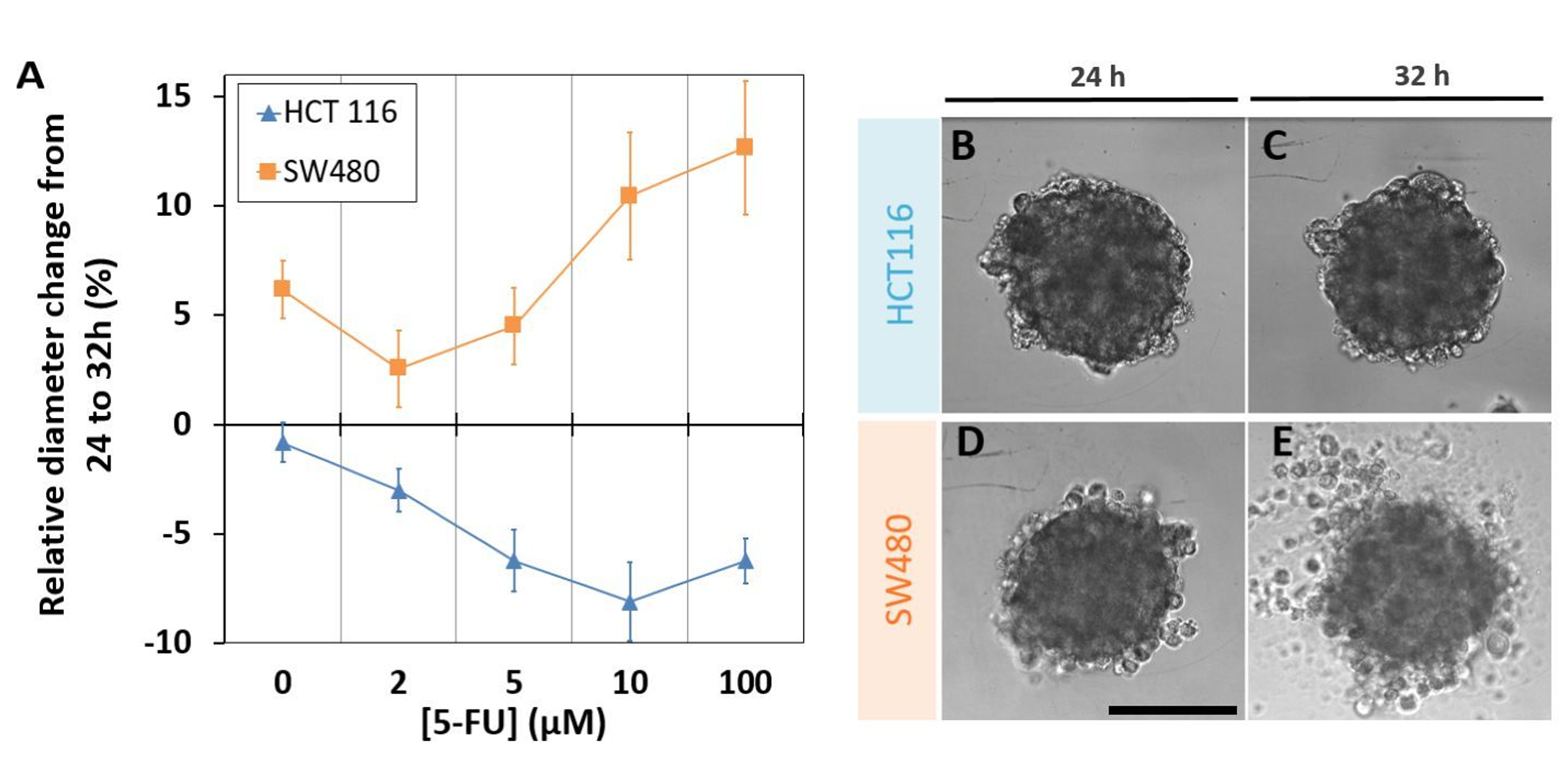

Supplement: S3 Fig — (A) Relative diameter change as a function of the 5-FU concentration. The diameter is evaluated from the spheroid surface area A including the diffuse outer layer measurement as (4A/π1/2. Error bars represent SEM (n = 7–12 for each cell line). (B,E) Typical images of MCTS at 24h (after transfer) and 32h for 10μM 5-FU. Scale bar, 200 μm. (TIF) [file pone.0188100.s003.tif]
